# Understanding Follicular Output Rate (FORT) and its Implications for POSEIDON Criteria

**DOI:** 10.3389/fendo.2019.00246

**Published:** 2019-04-16

**Authors:** Michael Grynberg, Julie Labrosse

**Affiliations:** ^1^AP-HP, Department of Reproductive Medicine & Fertility Preservation, Hôpital Antoine Béclère, Clamart, France; ^2^AP-HP, Department of Reproductive Medicine & Fertility Preservation, Hôpital Jean Verdier, Bondy, France; ^3^INSERM U1185, University Paris-Sud, Le Kremlin-Bicêtre, France; ^4^INSERM U1133, University Paris Diderot, Paris, France

**Keywords:** follicular output rate, FORT, POSEIDON criteria, hypo-response, controlled ovarian stimulation, FSH receptor polymorphism

## Abstract

The management of low prognosis patients in ART represents a challenge for reproductive specialists. Different profiles and biologic characteristics have been identified among these patients. Indeed, while poor ovarian response can be seen in patients with impaired ovarian reserve, others, identified as hypo-responders, show unexpected poor or suboptimal response to controlled ovarian stimulation despite satisfying ovarian parameters. These hypo-responders are associated during FSH stimulation to slow initial responses in terms of estradiol levels and follicle growth, longer stimulations, and/or greater cumulative FSH doses. Hence, it appears that ovarian sensitivity to gonadotropins differs from a patient to another, and plays a determinant role on ovarian response to stimulation. Although precise mechanisms remain to be elucidated, increasing evidence suggests that ovarian sensitivity to FSH could be influenced by the presence of genetic mutations or single nucleotide polymorphisms of gonadotropins and their receptors. Evaluating ovarian sensitivity to FSH therefore appears as a key element to improve IVF success rates in these low prognosis patients and open new treatment perspectives. Since the traditional ovarian markers currently used are not sufficient to accurately reflect ovarian response to FSH, a tool to assess ovarian sensitivity to gonadotropin stimulation was required. The present review aims to present Follicular Output Rate (FORT) as an efficient quantitative and qualitative marker of ovarian responsiveness to gonadotropins, discuss the underlying mechanisms of impaired sensitivity to FSH and the possible FORT implications for Poseidon criteria.

## Introduction

Mechanisms underlying poor ovarian response (POR) in assisted reproductive technology (ART) remain unclear. As no consensus on the management of poor responders exists, these low prognosis patients represent a challenge for reproductive specialists (1). The Bologna Criteria (2), established in 2011, defined poor responders to controlled ovarian stimulation (COS) by the presence of at least two of the following characteristics: advanced maternal age (≥40 years), a previous incident of POR (cycles canceled or ≤3 oocytes with a conventional ovarian stimulation protocol), and/or a low ovarian reserve tests [antral follicle count (AFC) <5–7 follicles or serum anti-Müllerian hormone (AMH) levels < 0.5–1.1 ng/mL]. Although successful in reducing the variability of POR definitions (3), the Bologna criteria failed to reflect the very different profiles and significantly variable biologic characteristics of these patients (4, 5). Notably, whereas POR can be observed in patients with impaired ovarian reserve, others show an “unexpected” poor or suboptimal response to COS despite satisfying ovarian parameters.

Consequently, the Poseidon classification (6) (with as endpoint the number of oocytes required to obtain at least one euploid embryo), distinguishes patients of low prognosis despite an adequate ovarian reserve (Groups 1 and 2: AFC >5 and AMH >1.2 ng/mL) from those with poor ovarian features (Groups 3 and 4: AFC <5 and AMH <1.2 ng/mL) (7). Patients of Poseidon Groups 1 and 2 show an initial slow response to FSH stimulation in terms of estradiol levels and follicle growth, require longer stimulations, and/or greater cumulative FSH doses despite their correct ovarian parameters (8, 9). Hence, markers currently used (such as AFC and AMH) are not sufficient to predict ovarian response accurately, notably for these “hypo-responders” who raise the question of ovarian sensitivity to FSH (10–12). Other methods are needed to enable identification and optimal counseling for these patients.

The present review aims to present Follicular Output Rate (FORT) as a tool to assess ovarian responsiveness to gonadotropins, discuss the underlying mechanisms of impaired sensitivity to FSH and the possible FORT implications for Poseidon criteria.

## Materials and Methods

A systematic search was led using the MEDLINE (PubMed), SCOPUS, EMBASE, and Cochrane Library databases, using the following keywords and MESH search terms: “follicular output rate,” “FORT,” “poor ovarian responder,” “poor ovarian response,” “POR,” “hypo-response,” “hypo-responder,” “ovarian sensitivity,” “Poseidon,” “assisted reproductive technology,” “ART,” “controlled ovarian stimulation,” “COS,” “COH,” “IVF,” “ICSI.” “FSH receptor,” “FSHR,” “polymorphism,” “LH receptor,” “LHR,” “pollution AND ovarian sensitivity.” All relevant studies (limited to human studies) published before October 2018 were considered, without language restrictions. The reference lists of relevant reviews and articles were also hand-searched.

## Follicular Output Rate (FORT)

So far, the strength of ovarian response to ovarian stimulation had been analyzed by considering the number of pre-ovulatory follicles obtained at the end of COS (13–16). However, the number of pre-ovulatory follicles obtained does not reliably reflect antral follicle responsiveness to FSH since it is greatly dependent on the number of pre-treatment small antral follicles (17). Similarly, the quantitative relationship between AMH levels and the number of mature follicles and fertilizable eggs observed in certain studies (18–22) may merely result from the positive correlation between AMH levels and pre-treatment number of small antral follicles (23–27), and does not itself attest of the sensitivity to FSH treatment (28). Therefore, identifying an index that considered the number of small antral pre-treatment follicles appeared crucial.

Genro et al. (28) were the first to introduce the concept of FORT in a prospective study of 162 patients. FORT was defined as the ratio of pre-ovulatory follicle (16–22 mm in diameter) count (PFC) on hCG day × 100/small antral follicle (3–8 mm in diameter) count at baseline. Patients (mean age of 34.6 ± 0.3 years) were undergoing COS protocol with a single-dose of time-release GnRH agonist on cycle days 1–3 (3 mg, IM, Decapeptyl, Ipsen Pharma, Paris, France), followed after complete pituitary desensitization had been confirmed, by daily recombinant FSH injections (Gonal-F, Serono Pharmaceuticals, Lyon, France) at a dosage of 300 IU/day for at least 5 days, and continued until the day of hCG. At baseline, women had 14.8 ± 0.3 antral follicles. After treatment, the total number of pre-ovulatory follicles obtained was 6.9 ± 0.2, with a corresponding FORT of 47.5 ± 1.4%. A positive relationship between serum AMH levels and the number of small antral (*p* < 0.0001) and PFC (*p* < 0.04) was observed. PFC tended to be lower in the low-AMH group when compared to the other groups (*p* = 0.246). Interestingly, FORT was negatively and significantly correlated with serum AMH levels, both in univariate and after stepwise regression analysis (*p* < 0.001). FORT values significantly and progressively decreased from the low (AMH < 1.69 ng/mL; *n* = 41), average (AMH 1.69–3.20 ng/mL; *n* = 82), to high (>3.20 ng/mL; *n* = 39) AMH groups. Whereas, FORT was positively associated to total recombinant FSH dose (*p* < 0.006) and duration of COS (*p* < 0.001), it was not significantly associated to age, body mass index, nor to basal estradiol or FSH levels.

Since then, FORT has been confirmed as an efficient quantitative, as well as qualitative, marker of ovarian response during COS. Gallot et al. (17) prospectively analyzed 322 patients who underwent the same protocol as that of Genro et al. (28). Patients were classified into three distinct FORT groups, arbitrarily chosen according to whether FORT values were under the 33th percentile (<42%, low FORT group; *n* = 102), between the 33th and the 67th percentile (42–58%, average FORT group; *n* = 123), or above the 67th percentile (>58%, high FORT group; *n* = 97) of distribution. Similarly, sets of three different groups according to ages, AFC, and PFC values were formed. Initial AFC was of 15.2 ± 0.2 follicles. Overall, FORT was of 50.6% (range, 16.7–100.0%). Coherently with previous results regarding the negative association between AMH levels and FORT (28), most patients (41%) of the low AFC group had a high FORT, while a minority of patients of the high AFC group (20%) belonged to the high FORT group. The number of oocytes and embryos obtained increased progressively from the low to the high FORT groups (*p* < 0.001), irrespective of age and absolute pre-COS AFC and post-COS PFC (Table 1). Furthermore, FORT levels were significantly correlated with the percentage of top-morphology embryos (*r* = 0.14; *p* < 0.02). Patients with a larger proportion of FSH-responsive antral follicles had better outcomes after IVF-ET, supporting the hypothesis that scant responsiveness of antral follicles to exogenous FSH reveals some degree of follicle/oocyte dysfunction (31, 32).

**Table 1:** Results of IVF-embryo transfer and outcome in the low, average, and high FORT groups.

	**Low FORT** **(<33th percentile)**	**Average FORT** **(33–67th percentile)**	**High FORT** **(>67th percentile)**	***p*-value**
**Gallot et al**. **(****17****)**				
Antral follicle count	16.6 ± 0.3	15.1 ± 0.4	14.0 ± 0.4	< 0.001
Pre-ovulatory follicle count	5.4 ± 0.1	7.5 ± 0.2	9.7 ± 0.3	< 0.001
Retrieved oocytes	8.6 ± 0.4	10.3 ± 0.4	11.8 ± 0.6	< 0.001
Number of metaphase II oocytes	7.4 ± 0.4	8.7 ± 0.3	10.0 ± 0.6	< 0.001
Total embryos	5.7 ± 0.3	5.9 ± 0.3	7.4 ± 0.4	< 0.002
Implantation rate (%)	23.3	34.4	37.7	< 0.004
Clinical pregnancies/oocyte retrieval (%)	33.3	51.2	55.7	< 0.004
Ongoing pregnancies/oocyte retrieval (%)	23.5	43.9	43.3	< 0.003
**Zhang et al**. **(****29****)**				
Antral follicle count	14.51 ± 6.33	14.00 ± 5.37	12.32 ± 4.28	< 0.001
Pre-ovulatory follicle count	5.49 ± 2.85	8.41 ± 3.27	11.54 ± 4.33	< 0.001
Retrieved oocytes	8.45 ± 5.64	11.52 ± 6.37	13.30 ± 6.34	< 0.001
Total embryos	4.94 ± 3.22	6.37 ± 3.69	7.33 ± 3.89	< 0.001
Good-quality embryo rate (%)	65.98	66.48	68.91	0.033
Implantation rate (%)	29.71	33.80	35.08	0.031
Clinical pregnancy rate (%)	46.27	53.80	55.86	0.012
**Hassan et al**. **(****30****)**				
Antral follicle count	16.7 ± 3.4	14.4 ± 4.5	11.4 ± 3.2	< 0.001
Pre-ovulatory follicle count	6.1 ± 1.6	7.6 ± 2.3	8.9 ± 2.4	< 0.001
Retrieved oocytes	5.4 ± 1.5	6.8 ± 2.3	7.4 ± 2.1	< 0.001
Number of metaphase II oocytes	4.5 ± 1.7	5.6 ± 2.4	6.2 ± 2.0	< 0.001
Fertilized oocytes	2.7 ± 1.5	3.9 ± 2.2	4.4 ± 1.9	< 0.001
Fertilization rate	48.4 ± 21.8	55.3 ± 20.3	57.4 ± 19.2	0.006
Total embryos	2.1 ± 1.3	3.2 ± 2.2	3.6 ± 1.8	< 0.001
Number of good-quality embryos	1.0 ± 1.0	2.0 ± 1.7	1.8 ± 1.4	< 0.001
Transferred embryos	1.4 ± 0.5	1.7 ± 0.4	1.8 ± 0.4	< 0.001
Clinical pregnancy rate (%)	29.9	43.3	57.8	< 0.001

Consistently, Zhang et al. (29) observed in a larger cohort of 1,503 non-PCOS patients that the number of retrieved oocytes and total number of embryos progressively increased from the low to high FORT groups (*p* < 0.001; Table 1). Mean FORT was of 65%. Moreover, Rehman et al. (33) found in a prospective study of 282 patients that an increase in FORT value by one unit was associated to increased mean numbers of oocytes retrieved (β coefficient: 0.135), metaphase II oocytes obtained (β coefficient: 0.128), and fertilized oocytes (β coefficient: 0.089). There was a positive relationship between FORT and clinical pregnancy rates (35.8%), and FORT values were higher in pregnant compared to non-pregnant patients (64.2 vs. 49.3%, respectively, *p* = 0.0001). Hassan et al. (30) reported similar results in a prospective study on 303 women undergoing IVF/ICSI for unexplained infertility. Patients were divided into three groups according to FORT: low FORT (*n* = 97), below the 33rd percentile, moderate FORT (*n* = 104) with values between the 33rd and the 67th percentiles, and high FORT (*n* = 102), above the 67th percentile. There was a progressive and significant increase from low to high FORT groups regarding number of retrieved oocytes (5.4 ± 1.5, 6.8 ± 2.8, and 7.4 ± 2.1, respectively; *p* < 0.001), clinical pregnancy rates (29.9, 43.3, and 57.8%, respectively; *p* < 0.001), and fertilization rates (48.4% ± 21.8 vs. 55.3% ± 20.3 and 57.4% ± 19.2, respectively; *p* = 0.006; Table 1). Multivariate logistic regression analysis revealed that the correlation between FORT and pregnancy was independent of potential confounding factors (*p* = 0.008).

## Mechanisms of Hypo-Response

Although ovarian hypo-response in ART remains to be elucidated, increasing evidence suggests that the presence of genetic mutations or single nucleotide polymorphisms (SNPs) of gonadotropins and their receptors could influence ovarian sensitivity to gonadotropin stimulation (8, 34, 35).

FSH receptor (FSH-R) is a type of G-protein-coupled receptor that mediates FSH intracellular signals through cyclic adenosine monophosphate pathways (8). Two polymorphisms of FSH-R (Thr307/Asn680 and Ala307/Ser680) have been associated to a higher requirement of exogenous gonadotrophins during COS (36, 37). Perez et al. (36) showed that basal FSH levels were significantly different according to FSH genotype (6.4 ± 0.4 IU/L, 7.9 ± 0.3 IU/L, and 8.3 ± 0.6 IU/L for Asn/Asn, Asn/Ser, and Ser/Ser groups, respectively, *p* < 0.01). The number of FSH ampoules required for successful stimulation was also significantly different among the three groups (31.8 ± 2.4, 40.7 ± 2.3, and 46.8 ± 5.0 for the Asn/Asn, Asn/Ser, and Ser/Ser groups, respectively, *p* < 0.05).

To better illustrate the relationship between FSH-R and FSH doses, Alviggi et al. (8) conducted a retrospective randomized study in which 17 patients requiring a cumulative dose of recombinant FSH (rFSH) >2,500 UI were compared to 25 patients requiring < 2,500 UI. Women requiring more than 2,500 UI of rFSH had significantly longer stimulations (*p* = 0.03), lower serum estradiol levels on hCG day (*p* = 0.001), a lower number of oocytes retrieved (*p* = 0.0005), and a lower number of transferred embryos (*p* = 0.001). Interestingly, the incidence of Ser/Ser genotype was higher in patients requiring greater doses of rFSH (*p* = 0.02). Also, patients with higher rFSH consumption and FSH-R Ser680 variant carriers had a longer infertility condition. Hence, FSH-R Ser680 may affect female fertility and delay pregnancy occurrence (8).

Moreover, the role of FSH polymorphisms on responsiveness to COS treatments was described in a meta-analysis of 33 studies lead by Alviggi et al. (38). Notably, the AA genotype of the FSH-R gene at position−29 has been reported to be associated with poor ovarian response. Achrekar et al. (39) showed in a retrospective analysis that subjects with AA genotype at the−29 position required higher amounts of exogenous FSH (*p* = 0.001), had significantly lower oestradiol concentrations before HCG day compared with the GA genotype (*p* = 0.015), and had a lower number of pre-ovulatory follicles (*p* = 0.001), and a lower number of retrieved oocytes (*p* = 0.003). Additionally, Desai et al. (40) observed that these AA genotype patients significantly expressed lower amounts of FSH-R protein, and that the relative mRNA expression of FSH-R was significantly decreased compared to GG genotype patients (*p* = 0.027). These results could be explained by the fact that DNA with the A allele might be less accessible for binding of transcription factors compared to the G allele.

However, polymorphisms of FSH-R do not seem to influence antral follicle responsiveness to strong FSH doses, as far as it is measurable by the FORT. Genro et al. (41) observed in 124 patients undergoing COS that FORT index were comparable between Thr307Ala and Asn680Ser carriers or non-carriers when stimulated with an initial dose of 300UI. Further studies using lower, yet more discriminating, FSH doses are required to determine whether this lack of difference is due to the intensity of the FSH signal or to a lack of functional relationship between these SNPs of FSHR and follicle reactivity to FSH.

The genotypic profile of LH receptors may also play a role in ovarian hypo-response. In the comparison of three groups undergoing a gonadotrophin-releasing hormone analog long protocol followed by stimulation with rFSH (Group A: 22 women requiring a cumulative dose of rFSH >3,500 IU; Group B: 15 patients requiring 2000–3500 IU; Group C: 23 women requiring < 2,000 IU), Alviggi et al. (42) showed that Group A had significantly lower estradiol peaks (*p* < 0.05) and a lower number of oocytes retrieved (*p* < 0.05) (7.3 ± 1.5, 11.7 ± 2.4, and 14.7 ± 4.1 in the three groups, respectively). Seven carriers (31.8%) of v-betaLH were found in Group A, whereas only one variant (6.7%) was observed in Group B and no variant was detected in Group C.

These results were confirmed in a larger series of 220 patients stimulated by rFSH (34). V-betaLH was present in 11% of patients. The study population was divided into two groups according to their LH genotype (wt/wt, *n* = 196; v-betaLH, *n* = 24). Patients with v-betaLH received a significantly higher cumulative-dose of r-hFSH (*p* = 0.048). LH genotype had a statistically significant effect on the cumulative dose of rFSH (*p* < 0.01), showing a progressive increase from wt/wt to v-betaLH heterozygotic and homozygotic women.

As few data exist on the potential influence of pollution on ovarian sensitivity, the role of environmental factors on ovarian response to COS remains to be elucidated. A Chinese study exploring the influence of fluoride exposure on FSH-R gene polymorphism in 679 women suggested that fluoride exposure was associated to lower GnRH serum levels, but no correlation with the FSH-R polymorphism AA genotype at position−29 was observed (43).

## Clinical Implications

Efficient as a quantitative and qualitative marker of ovarian sensitivity to FSH, FORT index should be used in everyday practice. Considering that only follicles between 16 and 22 mm on hCG day effectively respond to FSH may be a possible limitation of FORT. Smaller follicles might also present some degree of FSH responsiveness. However, as it is also possible that very small follicles, which could not be counted by ultrasound at baseline, may also have begun FSH-driven maturation after the start of COS and reached intermediate sizes on hCG day, the inclusion of average-sized follicles on hCG day into the calculation of FORT could confuse interpretation of the results. FORT is also limited by the technical impossibility to track the development of each follicle individually, and therefore cannot assess the possible differences in the FSH-driven growth of follicles (28).

Other tools such as Ovarian sensitivity index (OSI) (44) have also been suggested as a surrogate of AMH assay in predicting ovarian responsiveness to FSH in IVF. OSI corresponds to the total FSH dose administered divided by the number of retrieved oocytes. However, the interpretation of OSI is limited by the fact that it was obtained using a GnRH-agonist buserelin plus rFSH in a classical long protocol; hence, the correlation between OSI and AMH observed could differ in case of a different stimulation schedule or different drugs. More recently, authors introduced Follicle to Oocyte Index (FOI) (9), defined by the ratio between the total number of oocytes collected at the end of ovarian stimulation and the number of antral follicles available at the start of stimulation. FOI ≤ 50 was considered low. Trigger type and efficiency may influence FOI, and further studies are warranted to confirm the use of FOI as a marker of ovarian response.

Assessing ovarian sensitivity to FSH with FORT and understanding mechanisms behind hypo-response in ART opens new possibilities in the treatment of hypo-responders. Increasing FSH doses has been proposed, notably by Behre et al. (45), who evaluated its effect in patients with Ser680 polymorphism. Patients were randomly assigned to an FSH dose of 150 UI/day or 225 UI/day. The control group (Asn/Asn, *n* = 44) received a dose of 150 UI/day. Peak estradiol levels on hCG day were significantly lower for patients stimulated with 150 UI/day (*p* = 0.028). Increasing the FSH dose from 150 to 225 UI/day overcame the lower oestradiol response in women with Ser/Ser.

The benefit of adding LH in hypo-responders has also been explored (46, 47). Ferraretti et al. (48) randomized women showing a hyporesponsiveness to FSH into three groups, one receiving an increased dosage of FSH (*n* = 54), one receiving administered recombinant LH (rLH) in addition to the increased dose of FSH (*n* = 54), and another was given additional FSH and LH using hMG as a combined drug (*n* = 22). Addition of rLH significantly improved pregnancy, implantation, and live birth rates. Regarding LH doses, the randomization of 46 patients undergoing ovarian stimulation in two groups (supplementation with a daily rLH dose of 75 UI or 150 UI) showed a significant advantage for patients receiving 150 UI in terms of mean number of oocytes retrieved and percentage of mature oocytes, whereas these patients received a significantly lower mean number of rFSH vials (44.6 ± 7.4 vs. 36.1 ± 3.8) (49).

Moreover, older patients in ART may notably benefit from additional LH. On the one hand, the endocrine changes occurring with ovarian aging include an increase of serum FSH levels in the early follicular phase, which are not accompanied by an LH increase but by a progressive decrease of basal androgen levels. Follicular capacity to induce androstenedione synthesis after rFSH administration is reduced in older patients compared with younger reproductive-aged patients, whereas E_2_ secretion is preserved by increased aromatase function (50). In a study lead by Bosch et al. (50), whereas patients up to 35 years old (*n* = 380) did not appear to benefit from rLH, patients aged 36 to 39 years (*n* = 340) had significantly higher implantation rates (95%CI[1.04–2.33]) when rLH was added. Clinically higher although not significant ongoing pregnancy rates per started cycle (95% CI[0.93–2.38]) were observed. Consistently, Humaidan et al. (51) showed a significant benefit of exogenous LH supplementation for women aged above 35 years old in terms of implantation rates and significantly reduced total FSH consumption.

## Conclusion

Considering the lack of efficient tool to accurately evaluate ovarian hypo-response, FORT proves to be a relevant and crucial quantitative, and qualitative index that should be used in everyday practice for the care and management of hypo-responders in ART. Impaired sensitivity to FSH revealed by FORT should be considered in the decision of treatment protocol, gonadotropin, and stimulation doses to be used for hypo-responders. Improving follicular responsiveness to FSH may also be a key to ameliorate prognosis of POSEIDON groups 3 and 4 “expected” poor responders. Reconsidering criteria for COH cancellation based on the output of follicle response to exogenous FSH rather than on the absolute counting of follicles recruited by treatment should be discussed. It is expected that a better understanding of low prognosis patients undergoing ART will help improve individualized ovarian stimulation management and identify more homogeneous populations for clinical trials, thereby, providing better tools with which to maximize IVF success rates.

## Author Contributions

All authors listed have contributed to the work and approved the final version. JL and MG performed the literature research, wrote the paper and proofread it.

### Conflict of Interest Statement

The authors declare that the research was conducted in the absence of any commercial or financial relationships that could be construed as a potential conflict of interest.
